# *Asxl2^−/−^* Mice Exhibit De Novo Cardiomyocyte Production during Adulthood

**DOI:** 10.3390/jdb4040032

**Published:** 2016-11-03

**Authors:** Rachel Brunner, Hsiao-Lei Lai, Zane Deliu, Elan Melman, David L. Geenen, Q. Tian Wang

**Affiliations:** 1 Department of Biological Sciences, University of Illinois at Chicago, Chicago, IL 60607, USA; rbrunn2@uic.edu (R.B.); hsiaoleilai@gmail.com (H.-L.L.); zdeliu2@uic.edu (Z.D.); emelma2@illinois.edu (E.M.); 2 PTM Biolabs Inc., Chicago, IL 60612, USA; 3 The School of Molecular and Cellular Biology, University of Illinois Urbana-Champaign, Champaign, IL 61801, USA; 4 Department of Physiology and Biophysics, University of Illinois at Chicago, Chicago, IL 60612, USA; geenend@gvsu.edu; 5 Physician Assistant Studies, Grand Valley State University, Grand Rapids, MI 49503, USA; 6 Congressionally Directed Medical Research Programs, Frederick, MD 21702, USA

**Keywords:** heart, cardiogenic, cardiomyocyte, proliferation, regeneration, chromatin factor, epigenetic

## Abstract

Heart attacks affect more than seven million people worldwide each year. A heart attack, or myocardial infarction, may result in the death of a billion cardiomyocytes within hours. The adult mammalian heart does not have an effective mechanism to replace lost cardiomyocytes. Instead, lost muscle is replaced with scar tissue, which decreases blood pumping ability and leads to heart failure over time. Here, we report that the loss of the chromatin factor ASXL2 results in spontaneous proliferation and cardiogenic differentiation of a subset of interstitial non-cardiomyocytes. The adult *Asxl2^−/−^* heart displays spontaneous overgrowth without cardiomyocyte hypertrophy. Thymidine analog labeling and Ki67 staining of 12-week-old hearts revealed 3- and 5-fold increases of proliferation rate for vimentin^+^ non-cardiomyocytes in *Asxl2^−/−^* over age- and sex-matched wildtype controls, respectively. Approximately 10% of proliferating non-cardiomyocytes in the *Asxl2^−/−^* heart express the cardiogenic marker NKX2-5, a frequency that is ~7-fold higher than that observed in the wildtype. EdU lineage tracing experiments showed that ~6% of pulsed-labeled non-cardiomyocytes in *Asxl2^−/−^* hearts differentiate into mature cardiomyocytes after a four-week chase, a phenomenon not observed for similarly pulse-chased wildtype controls. Taken together, these data indicate de novo cardiomyocyte production in the *Asxl2^−/−^* heart due to activation of a population of proliferative cardiogenic non-cardiomyocytes. Our study suggests the existence of an epigenetic barrier to cardiogenicity in the adult heart and raises the intriguing possibility of unlocking regenerative potential via transient modulation of epigenetic activity.

## 1. Introduction

Heart disease is the leading cause of death in developed countries. Many forms of heart disease result in a loss of functional muscle. Unfortunately, the heart’s natural ability to generate new muscle is severely limited. The development of effective therapies for cardiac regeneration will benefit from a thorough understanding of endogenous cardiogenic mechanisms and how they are regulated.

Very limited cardiomyocyte production occurs in the adult heart and past research has reported two mechanisms by which this occurs. The most indisputable mechanism is renewal via cardiomyocyte proliferation [1,2,3,4,5,6,7,8,9]. Genetic fate-mapping studies suggest that this is the dominant mechanism for cardiomyocyte production in the adult mammalian heart [5,7,8,10]. However, the majority of studies estimate the rate of cardiomyocyte proliferation to be very low during adulthood, at or below ~1% per year [1,2,4,5,6,7,8,9]. It is possible to stimulate proliferation by manipulating the activity of certain genes [11,12,13,14,15,16,17,18,19,20,21,22,23,24].

The second mechanism of cardiomyocyte production in the adult heart involves the differentiation of resident cardiac cells with progenitor activity [25]. For example, c-kit^+^ cardiac stem cells (CSCs) have been reported to produce cardiomyocytes in the adult heart [26]. However, the estimated efficiency of this process is drastically different between different studies [27,28,29,30]. The adult epicardial cells have also been reported to produce cardiomyocytes after thymosin β4 treatment in the normal or infarcted adult heart [31,32,33], though an effort to duplicate this result was unsuccessful [34]. Mechanisms for cardiomyocyte production in the adult heart have been identified, however, a better understanding of their regulation will help pave the way to develop therapies to replace lost muscle tissue. 

Aside from cardiomyocyte proliferation and cardiogenic differentiation from CSCs, cardiomyocytes can be derived from several populations of cells isolated from the adult heart, such as Sca1^+^ cells [35], side population (SP) cells [36], Isl1^+^c-kit^+^ cells [37], and cardiac colony-forming units-fibroblast (cCFU-Fs) [38]. It is unclear whether these cells are normally cardiogenic in vivo or if cardiogenicity was induced by in vitro culturing. 

Finally, cardiac fibroblasts (CFs) can be reprogrammed into cardiomyocytes both in vitro and in vivo through the transduction of a cocktail of three transcription factors, GATA4, MEF2C, and TBX5 [39,40,41]. This mechanism does not appear to be active in the normal adult heart, as genetic tracing of the CF lineage using Periostin (Postn)-Cre did not find sign of spontaneous trans-differentiation of CFs into cardiomyocytes [41,42].

Here we report de novo cardiomyocyte production at a significant rate in adult mice carrying a mutation in *Asxl2*, a chromatin-associated factor. The hearts of *Asxl2^−/−^* mice exhibit significant growth between two and four months after birth. This growth is not due to cardiomyocyte hypertrophy. Rather, we present data that the adult *Asxl2^−/−^* heart harbors a population of proliferative interstitial cells that undergo spontaneous cardiogenic differentiation. 

We have previously shown that ASXL2 is an important regulator of histone H3 methylation and H2A deubiquitination [43,44]. The implication of ASXL2 in the regulation of cardiomyocyte production during adult life raises new therapeutic possibilities for cardiac repair and regeneration.

## 2. Materials and Methods 

### 2.1. Animals

*Asxl2* mutant mice were generated by utilizing a gene-trapped embryonic stem cell line from the Gene-Trap Consortium (http://www.genetrap.org/) [43]. The *Asxl2^−^* allele yields an mRNA with the first 19 amino acids of ASXL2 followed by the gene trap cassette. The resultant fusion protein contains none of the conserved domains of ASXL2.

The *Asxl2^−^* allele is currently in two inbred genetic backgrounds, C57BL/6J and 129/Sv. Viable homozygous mutants (*Asxl2^−/−^*) are not recovered in either inbred genetic background, but are recovered when heterozygous C57BL/6J mice are mated to heterozygous 129/Sv mice. Experiments presented here are from such F1 progeny.

All animal studies were performed in accordance with the University of Illinois at Chicago Institutional Animal Care and Use Committee (IACUC) and Animal Care Committee policies (ACC 13-117).

### 2.2. Assessment of Heart Growth

Transthoracic echocardiography and left ventricular (LV) mass calculation were performed as previously described [45,46]. LVMI is LV mass (mg) expressed relative to body mass (g). 

Direct measurement of heart weight was performed with freshly dissected hearts that had been trimmed of excess large vessel tissue and gently blotted. Heart mass index is the heart mass (mg) expressed relative to body mass (g).

Quantitative morphometric analysis of total ventricular muscle volume was performed using a previously described method for calculating scar volume [5], with some modifications. Paraffin embedded hearts from wildtype and *Asxl2^−/−^* animals at 8- and 16-week of age were cross-sectioned (5 μm). Whole hematoxylin and eosin stained sections at 125 μm intervals were imaged at 10× on a Zeiss Observer.Z1 with ZenPro software using the tiling feature. The area covered by the section was calculated using ImageJ, then multiplied by the interval of 125 μm, and these volumes were totaled to give the ventricle muscle volume per heart. To normalize for differences in body mass, ventricle muscle volume (mm^3^) is expressed relative to body mass (g).

### 2.3. Morphometric Analysis of Isolated Cardiomyocytes

Adult cardiomyocytes were isolated as previously described [47]. Glutaraldehyde fixed isolated cardiomyocytes from 8- and 16-week wildtype and *Asxl2^−/−^* hearts were analyzed by Martin Gerdes’ Lab (New York Institute of Technology) to determine the following: cell volume (Coulter Counter/Channelyzer), cell length and cell profile area (microscopically by image analysis), cross-sectional area (calculated from cell volume/cell length), and nucleation status (Figure 2 and Supplemental Figure S3).

Cardiomyocytes were also isolated from hearts of wildtype and *Asxl2^−/−^* that were pulse-labeled with EdU at 12-weeks, and chased 4-weeks (Figure 6). Isolated cardiomyocytes were fixed in 4% paraformaldehyde for 10 min on ice with frequent agitation to prevent aggregation of the cardiomyocytes, permeabilized with 0.5% Tritonx-100 in PBS for 10 min, and then labeled for EdU incorporation (Click-iT^®^ EdU Imaging Kit, Invitrogen, Waltham, MA, USA, C10337), per the manufacturer’s instructions. Cardiomyocytes were then co-labeled with mouse-α-cTnT (Thermo Scientific, Waltham, MA, USA, MS-295), labeled with biotin-conjugated anti-mouse IgG (Vector, M.O.M Kit, BMK-2202), and subsequently labeled with streptavidin conjugated to AlexaFluor 594 (Jackson ImmunoResearch, 016-580-084). The cardiomyocytes were re-suspended in mounting medium (Vectashield with DAPI (4′,6-diamidino-2-phenylindole), Vector Laboratories, H-1200).

Imaging was performed at the Northwestern University Center for Advanced Microscopy (generously supported by CCSG P30 CA060553 awarded to the Robert H Laurie Comprehensive Cancer Center). Cardiomyocytes were visualized using a Zeiss upright AXIO microscope at 10× magnification. The Tissue Gnostics system (Vienna, Austria) and Tissue FAXs software were utilized to image entire slides, with the individual tiles (defined as one 10× image field) being exported for analysis. Each tile was manually analyzed for cardiomyocytes with EdU^+^ nuclei. To estimate the total number of cardiomyocytes, they were manually counted in every tenth tile. These numbers were then averaged per slide, and subsequently multiplied by ten to give the estimated total number of cardiomyocytes per slide. From these data, the percentage of EdU^+^ labeled cardiomyocytes among all analyzed isolated cardiomyocytes from chased hearts was calculated. 

To analyze the morphology and nucleation status of EdU^+^ cardiomyocytes, the 20× objective and ZenPro software was used to acquire images of EdU^+^ cardiomyocytes. All imaged cardiomyocytes were in a longitudinal orientation, had smooth membranes, and sarcomeres were clearly evident. A total of 70 EdU^+^ cardiomyocytes (from two *Asxl2^−/−^* hearts) were analyzed. The nucleation status (mono- or bi-nucleated; EdU^+^ cardiomyocytes with more than two nuclei were not observed) was recorded for each EdU^+^ cardiomyocyte. Additionally, the length of each EdU^+^ cardiomyocyte was measured using Pixel Stick (Plum Amazing). From this analysis, EdU^+^ cardiomyocytes were placed into one of three categories: (1) mononuclear and shorter than the shortest cardiomyocytes observed from four-month-old wildtype hearts; (2) mononuclear and within range of lengths of cardiomyocytes from four-month-old wildtype hearts; or (3) binuclear and within range of lengths of cardiomyocytes from four-month-old wildtype hearts.

### 2.4. Thymidine Analog Pulse and Pulse-Chase Assays

Mice were assayed for proliferation at two time-points, week 8 and week 12. Mice were injected with a thymidine analog, either 100 mg/kg 5-bromo-2-deoxyuridine (BrdU, Sigma, St. Louis, MO, USA, B5002) or 50 mg/kg 5-Ethynyl-2′-deoxyuridine (EdU, Invitrogen, A10044), once daily for three consecutive days. Hearts were harvested approximately 6 h post the final injection. 

Paraffin sections were labeled for EdU or BrdU, imaged, and analyzed for proliferation (for details see methods section on “Immunofluorescence” below). BrdU^+^ or EdU^+^ nuclei were counted manually and total nuclei per image was found using Cell Profiler software [48]. The proliferation index is expressed as the percentage of BrdU^+^ nuclei of total nuclei.

To determine the fate of cells that proliferated at 12-week, mice were injected with EdU at 12 weeks of age (as above), and hearts were harvested 4 weeks after the final EdU injection, allowing time for the newly proliferated to differentiate. 

### 2.5. Preparation of Histological Sections

Prior to harvest, mice were injected with heparin, then euthanized with carbon dioxide, followed by cervical dislocation. Hearts were extracted and submerged in 1 M potassium chloride (in phosphate buffered saline). Hearts were then trimmed to remove lung and large vessel tissues, gently blotted dry, and weighed. 

Hearts to be paraffin-embedded were fixed in cold 4% paraformaldehyde overnight. Hearts were then washed in PBS and a small amount of tissue was cut from the dorsal and ventral sides of the heart to expose the left and right ventricles to facilitate infiltration (this step was omitted for hearts used for quantitative morphometric ventricle volume analysis). Hearts were dehydrated through a graded series of isopropanol (Sigma, St. Louis, MO, USA, 534021-4L) in 20 min/10 mL washes as follows: 50%, 75%, 95%, and 100% twice. Isopropanol was dissolved in dH_2_O and samples were shaken gently on a horizontal rotator in six-well plates. Hearts were cleared with mineral oil (Sigma, M8410) as follows, 25%, 50%, 75% (mixed with isopropanol), and three changes of 100% mineral oil (using the same conditions as dehydration steps). The hearts were then infiltrated with paraffin (McCormick Scientific, St. Louis, MO, USA, 39502004) as follows: 25%, 50%, 75% (dissolved in mineral oil, 20 min, 59 °C), and followed by two changes in 100% paraffin. Samples were then positioned in molds and allowed to cool to room temperature. Five-micron sections were cut, dried vertically overnight, and heat-fixed to the slides the following day for 2 h at 42 °C.

For frozen sections, freshly dissected, and trimmed hearts were placed in optimum cutting temperature (O.C.T.) medium (TissueTek, 4583) and snap-frozen in a hexane/dry ice bath. Five-micron sections were cut on a cryotome, mounted on charged slides, and stored at −20 °C until use.

### 2.6. Immunofluorescence

For immunofluorescent analysis of paraffin sections, slides were de-paraffinized in xylene and rehydrated through a graded series of ethanol. Antigen unmasking was performed with 10 mM Tris, 1 mM EDTA, 0.05% Tween-20, pH 9.0 at 92 °C for 20 min. Sections were then permeabilized with 0.2% TritonX-100 (in PBS) for 10 min. Blocking was done using 5% normal serum (in PBS) of the species the secondary antibody was raised in. EdU incorporation was detected using a Click-iT^®^ EdU Imaging Kit (Invitrogen, C10337), per manufacturer’s instructions.

Sections were incubated in primary antibody overnight at 4 °C in a humidified chamber. Primary antibodies used on paraffin sections include rat-α-BrdU (1:100, Abcam, Cambridge, MA, USA, ab6326), mouse-α-cTnT (1:100, Thermo Scientific, MS-295), rabbit-α-Ki67 (1:400, Abcam, ab15580), rabbit-α-vimentin (1:100, Abcam, ab92547), rabbit-α-CD31 (1:40, Lifespan BioSciences, Seattle, WA, USA, LS-B1932), rabbit-α-α-smooth muscle actin (1:800, Sigma, A2547), rabbit-α-NKX2-5 (1:100, Abcam, ab22611), rabbit-α-MEF2C (1:100, Abcam, ab64644), rabbit-α-GATA4 (1:100, Santa Cruz, Dallas, TX, USA, sc-9053), and rabbit-α-connexin43 (1:200, Santa Cruz, sc-6560). Cell membranes were labeled with wheat germ agglutinin (WGA) conjugates, either WGA conjugated to fluorescein isothiocyanate (FITC) (20 μL/mL, Vector, FL-1021) or WGA-CF™ 594 conjugate (1:200, Biotium, Fremont, CA, USA, 29023-1). All secondary antibodies were from Jackson ImmunoResearch and used at a concentration of 1:200. All nuclei were labeled either with DAPI (Vectashield + DAPI mounting media, Vector Laboratories, Burlingame, CA, USA, H-1200) or Hoechst (1:2000, Invitrogen). If Hoechst was used, slides were mounted with Vectashield (Vector Laboratories, H-1000).

For immunofluorescence on frozen sections, sections were fixed with methanol. After fixation, sections were incubated in primary antibody overnight at 4 °C in a humidified chamber. Primary antibodies used on frozen sections include rabbit-α-C-Kit (Abcam, ab5506) and rat-α-PDGFRα (BD Pharmingen, 558774). 

Sections were imaged using a Zeiss Axiovert 200 M or a Zeiss Observer.Z1 microscope using the 20× objective. Twenty-five images were taken per frontal section as follows: 10 images of the left ventricular free wall (five images each of epicardial side and endocardial side), 10 images of the septum (five images each of the left and right ventricular sides) and five images of the right ventricular free wall.

## 3. Results

### 3.1. Overgrowth of the Adult Asxl2^−/−^ Heart without Cardiomyocyte Hypertrophy

**Figure 1:**
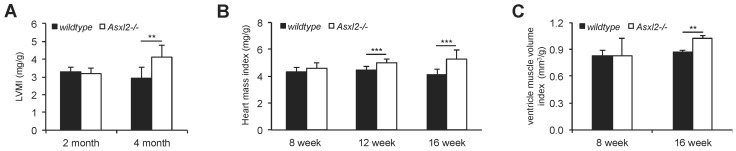
Overgrowth of the adult *Asxl2^−/−^* heart. (**A**) Left ventricular mass index found by echocardiography at 2-month (wildtype, *n* = 4; *Asxl2^−/−^*, *n* = 4) and 4-month (wildtype, *n* = 7; *Asxl2^−/−^*, *n* = 7) of age, normalized to body mass; (**B**) Mass of freshly dissected hearts normalized to body mass at 8-week (wildtype, *n* = 6; *Asxl2^−/−^*, *n* = 6), 12-week (wildtype, *n* = 16; *Asxl2^−/−^*, *n* = 11), and 16-week (wildtype, *n* = 12; *Asxl2^−/−^*, *n* = 9) of age; (**C**) Quantitative morphometric analysis of ventricle muscle volume normalized to body mass at 8-week (wildtype, *n* = 3; *Asxl2^−/−^*, *n* = 3) and 16-week (wildtype, *n* = 3; *Asxl2^−/−^*, *n* = 3) of age. Bars indicate standard deviation. *p-*Values (Student’s *t-*test): ** <0.01; *** <0.005.

Our previously published results suggested that *Asxl2^−/−^* animals have enlarged hearts [43]. To further investigate this phenotype, we took three approaches to evaluate the growth of *Asxl2^−/−^* and wildtype hearts: echocardiographic measurement of heart dimensions in live animals, weight measurement of freshly dissected hearts, and quantitative morphometric analysis of paraffin embedded hearts. All three approaches showed that from approximately eight weeks onwards, the rate of cardiac growth in *Asxl2^−/−^* mice outpaced the rate of body weight growth (Figure 1, Supplemental Figures S1 and S2); in contrast, the rate of cardiac growth in wildtype mice either parallels or is slower than that of body weight growth depending on the means of measurement (Supplemental Figure S1). By 12 weeks, the hearts of *Asxl2^−/−^* animals are disproportionally larger than those of wildtype controls.

**Figure 2:**
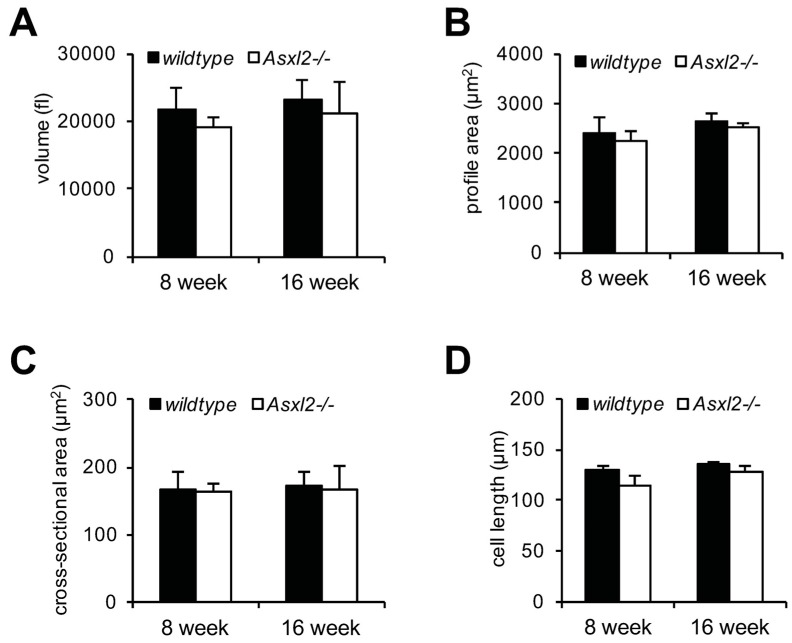
Lack of cardiomyocyte hypertrophy in *Asxl2^−/−^* heart. Isolated cardiomyocytes from 8- and 16-week old wildtype and *Asxl2^−/−^* hearts were assessed for: (**A**) volume; (**B**) profile area; (**C**) cross-sectional area; and (**D**) length. Sample size: 8-week wildtype, *n* = 4; 8-week *Asxl2^−/−^*, *n* = 3; 16-week wildtype, *n* = 5; 16-week *Asxl2^−/−^*, *n* = 3. Bars indicate standard deviation. No significant differences were observed (Student’s *t*-test).

Growth of the normal adult heart is predominantly the result of growth in size of existing cardiomyocytes [49,50]. We isolated cardiomyocytes from *Asxl2^−/−^* and wildtype hearts and assessed nucleation, cell dimensions, and volume (using the high-throughput Coulter Counter/Channelyzer method [51]). *Asxl2^−/−^* cardiomyocytes are not larger than wildtype nor is there a higher percentage of binucleated cardiomyocytes at either 8- or 16-week of age (Figure 2 and Supplemental Figure S3). In fact, while not statistically significant, *Asxl2^−/−^* cardiomyocytes appear to be somewhat smaller than wildtype (Figure 2). These observations are consistent with a previous imaging-based measurement showing that *Asxl2^−/−^* cardiomyocytes are not hypertrophic at six months of age [45]. We conclude that the overgrowth of the *Asxl2^−/−^* heart observed after approximately eight weeks cannot be accounted for by cardiomyocyte hypertrophy.

### 3.2. Asxl2^−/−^ Hearts Exhibit Elevated Proliferative Activity, but Not in Cardiomyocytes 

**Figure 3:**
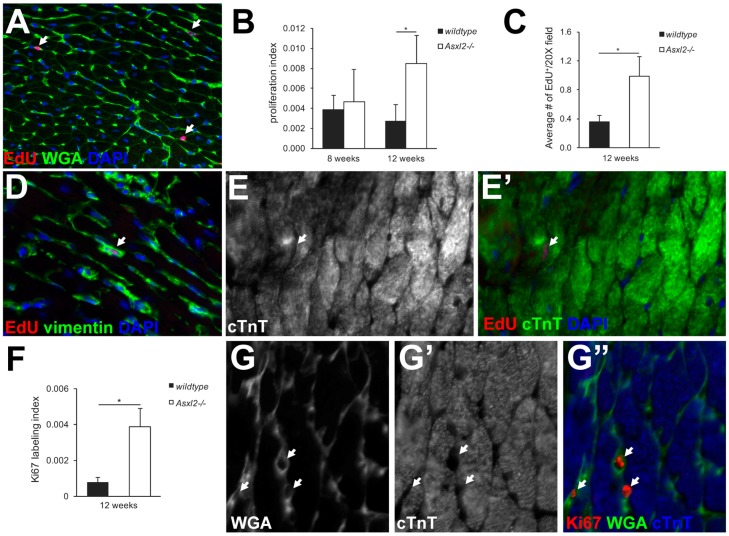
The *Asxl2^−/−^* hearts exhibit elevated proliferative activity in non-cardiomyocytes. (**A**) Representative image showing EdU, wheat germ agglutinin (WGA), and 4′,6-diamidino-2-phenylindole (DAPI); (**B**) Quantification of BrdU^+^ nuclei in left ventricle (LV) at 8-weeks (wildtype: *n* = 3, 3 non-consecutive sections/heart, 116 BrdU^+^, 30,045 total nuclei; *Asxl2^−/−^*: *n* = 3, 3 non-consecutive sections/heart, 170 BrdU^+^, 36,564 total nuclei) and 12-weeks (wildtype: *n* = 3, three non-consecutive sections/heart, 35 BrdU^+^, 13,853 total nuclei; *Asxl2^−/−^*: *n* = 3, three non-consecutive sections/heart, 127 BrdU^+^, 15,250 total nuclei); (**C**) Quantification of average number of EdU^+^ per 20× image field at 12-weeks (wildtype: *n* = 3, three non-consecutive sections/heart, 75 images; *Asxl2^−/−^*: *n* = 3, three non-consecutive sections/heart, 75 images); (**D**) Representative image of EdU, vimentin and DAPI labeling; (**E**,**E′**) Representative image of EdU, cTnT and DAPI labeling; (**F**) Quantification of Ki67^+^ nuclei in LV at 12-weeks (wildtype: *n* = 3, three non-consecutive sections/heart, 51 Ki67^+^, 67,727 total nuclei; *Asxl2^−/−^*: *n* = 3, three non-consecutive sections/heart, 236 Ki67^+^, 62,124 total nuclei); (**G**–**G″**) Representative image of Ki67, WGA and cTnT labeling. Arrows indicate proliferative cell nuclei. Bars indicate standard deviation. * *p*-Value (Student’s *t*-test) < 0.05.

The mutation or transgenic expression of certain genes can induce elevated proliferation of cardiomyocytes in the adult heart, leading to heightened heart growth [11,12,13,15,16,17,18,19]. To determine whether this is the case for *Asxl2^−/−^* hearts, we labeled proliferating cells using thymidine analogs (BrdU or EdU) (Figure 3A–E) and Ki67 (Figure 3F–G). BrdU labeling revealed a ~3-fold higher proliferation index in the *Asxl2^−/−^* left ventricle at 12-weeks (*p*-value = 0.042, Student’s *t*-test; Figure 3B). EdU labeling gave comparable results (Figure 3C). A similar trend was observed by Ki67 staining; ~5-fold more Ki67^+^ nuclei were found in the *Asxl2^−/−^* left ventricle compared to the wildtype (*p*-value = 0.028, Student’s *t*-test; Figure 3F). Thus, both methods showed an elevated proliferative activity in the *Asxl2^−/−^* hearts during the time window of heart enlargement. However, very few of the BrdU-, EdU-, or Ki67-labeled cells appeared to be cardiomyocytes. In both *Asxl2^−/−^* and wildtype hearts, the vast majority of EdU^+^ cells were interstitial (98.6% ± 1.2% in *Asxl2^−/−^*, compared to 98.7% ± 0.4% in wildtype) and cTnT^−^ (97.4% ± 2.4% in *Asxl2^−/−^*, compared to 98.1% ± 1.3% in wildtype) (Figure 3A,E). Almost all EdU^+^ cells were vimentin^+^ (97.5% ± 3.2% in *Asxl2^−/−^*, compared to 98.3% ± 1.8% in wildtype) (Figure 3D, Supplemental Figure S4A,B), and ~25% were CD31^+^ (Supplemental Figure S4C,D). Similar observations were made on Ki67-stained hearts (Figure 3G).

### 3.3. Expression of Cardiogenic Markers by Proliferative Cells in Asxl2^−/−^ Hearts

**Figure 4:**
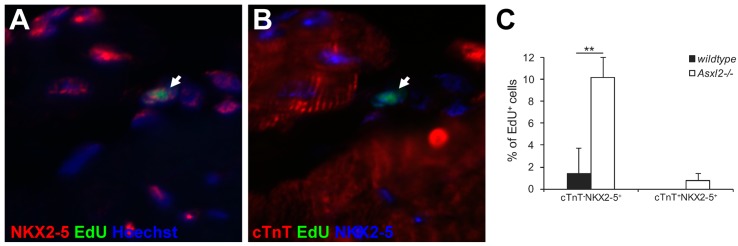
EdU^+^ cells in 12-week *Asxl2^−/−^* hearts show signs of being cardiogenic. (**A**,**B**) Paraffin heart sections from EdU-treated 12-week wildtype and *Asxl2^−/−^* animals were labeled for EdU, NKX2-5, and cTnT; (**C**) Quantification of the percentage of EdU-labeled cells that were EdU^+^NKX2-5^+^cTnT^−^ and EdU^+^NKX2-5^+^cTnT^+^. Sample size: *n* = 3 animals per genotype; three non-consecutive sections/heart; analyzed fifteen 20× left ventricle images/section; 177 wildtype EdU^+^ cells, 252 *Asxl2^−/−^* EdU^+^ cells. Bars represent standard deviation. ** *p-*Value (Student’s *t*-test) < 0.01.

Cardiomyocytes are by far the largest cells in the heart. The bulk of the size and weight of the heart comes from cardiomyocytes [52]. The moderate over-proliferation of non-cardiomyocytes in *Asxl2^−/−^* hearts is unlikely sufficient to cause an apparent increase in heart size, unless some proliferating cells are cardiogenic and give rise to cardiomyocytes. We pulse labeled proliferating cells in 12-week-old *Asxl2^−/−^* hearts by EdU or BrdU and asked whether labeled cells express NKX2-5, MEF2C, or GATA4, three transcription factors associated with cardiogenicity. Among all EdU^+^cTnT^−^ cells, the percentage of NKX2-5^+^ cells was ~7-fold higher in *Asx2^−/−^* hearts compared to wildtype (*p*-Value = 0.008, Student’s *t*-test; Figure 4; distribution shown in Supplemental Figure S5). The percentage of MEF2C^+^ cells was ~2.9-fold higher (*p* = 0.018; Supplemental Figure S6A,B). Very few EdU^+^GATA4^+^ cells and no EdU^+^ISL1^+^ were observed in either genotype (Supplemental Figure S6C,D and data not shown).

### 3.4. EdU-Labeled Cells Give Rise to Cardiomyocytes in Asxl2^−/−^ Hearts after 4-Week Chase

**Figure 5:**
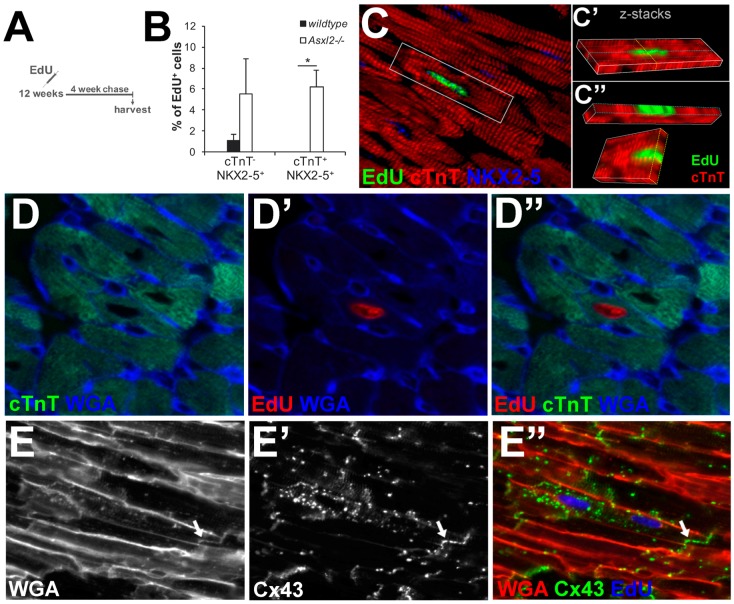
EdU-labeled cells give rise to cardiomyocytes in *Asxl2^−/−^* hearts after 4-week chase. (**A**) Schematic of the lineage tracing assay used to assess the differentiation potential of the proliferating non-cardiomyocytes observed at 12 weeks of age; (**B**) Quantification of the percentage of EdU^+^ cells that are cTnT^−^NKX2-5^+^ and cTnT^+^NKX2-5^+^ in the left ventricle following the chase; (**C**) Representative image of a mononuclear, EdU^+^cTnT^+^NKX2-5^+^ cell in a longitudinal orientation; (**C′**) The same cell in c, shown in a z-stack image; (**C′′**) Cross-sections of the z-stack image in c′; (**D**–**D′′**) Representative image of an EdU^+^cTnT^+^ cell, delineated with wheat germ agglutinin (WGA), in a transverse heart section; (**E**–**E′′**) Representative image of EdU, WGA and labeling for the gap junction protein, connexin 43 (CX43). The arrow indicates the CX43^+^ membrane between two cardiomyocytes. Sample size: *n* = 3 animals per genotype; three non-consecutive sections/heart; analyzed LV from whole section-stitched images; 617 and 755 EdU^+^ cells were examined on wildtype and *Asxl2^−/−^* heart sections, respectively. Bars represent standard deviation. * *p-*Value (Student’s *t*-test) < 0.05.

To definitively test whether the proliferative cells in *Asxl2^−/−^* hearts produce de novo cardiomyocytes, we pulse labeled *Asxl2^−/−^* and control hearts with EdU at 12-weeks and examined the fate of EdU-labeled cells after a 4-week chase (Figure 5A). While there is limited knowledge on the elimination half-life of EdU, for the closely related BrdU it is ~15–30 min [53,54]. Therefore, we expect that all cells that took up EdU did so during the pulse-labeling period and not during the chase. Cardiomyocytes were identified by markers (cTnT and Nkx2.5 staining) and by cell size (WGA staining). After the chase, there was a significant increase in the percentage of EdU^+^cTnT^+^NKX2-5^+^ cells (*p*-value = 0.018, Student’s *t*-test) in *Asxl2^−/−^* and a concomitant trend toward a decrease in the percentage of EdU^+^cTnT^−^NKX2-5^+^ cells, but not in wildtype hearts (Figure 4C and Figure 5B). EdU^+^ cardiomyocytes were observed readily in chased *Asxl2^−/−^* hearts (Figure 5C–E), but not in chased wildtype hearts. EdU^+^NKX2-5^+^cTnT^+^ cells represented 6.2% ± 1.6% of all EdU^+^ cells in the chased *Asxl2^−/−^* left ventricle (Figure 5B). The gap junction protein connexin 43 (Cx43) was detected on the membrane between EdU^+^ cardiomyocytes and neighboring EdU^−^ cardiomyocytes (Figure 5E), suggesting electrical coupling between the two. Concomitant to the appearance of EdU^+^ cardiomyocytes in *Asxl2^−/−^* hearts after the chase, there was a significant decrease of EdU^+^vimentin^+^ cells (Supplemental Figure S4B). In contrast to EdU^+^cTnT^+^NKX2-5^+^ cells, the percentage of EdU^+^CD31^+^ or EdU^+^α-SMA^+^ cells did not increase after the chase period (Figure 5B and Supplemental Figure S4C–F), suggesting that there was no significant production of endothelial or smooth muscle cells. Finally, chased wildtype hearts showed neither appearance of EdU^+^ cardiomyocytes nor decrease of EdU^+^vimentin^+^ cells (Figure 5B, Supplemental Figure S4B). Taken together, these data suggest that a significant number of proliferating interstitial cells differentiate into cardiomyocytes in the *Asxl2^−/−^* heart, but not in the wildtype heart.

**Figure 6:**
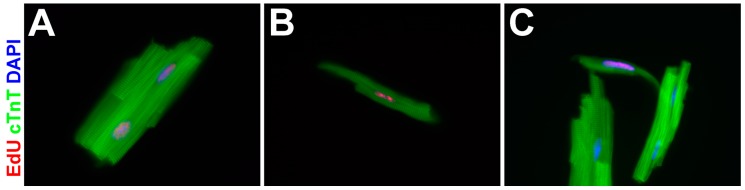
*Asxl2^−/−^* EdU^+^ cardiomyocytes display a range of size, morphology, and nucleation status. Cardiomyocytes were isolated and stained for EdU, cTnT, and DAPI. Representative images highlighting differences among *Asxl2^−/−^* EdU^+^ cardiomyocytes shown are (**A**) binuclear and rod-shaped with length comparable to EdU^−^ cardiomyocytes; (**B**) mononuclear and rod-shaped with normal length; and (**C**) mononuclear, spindle-shaped, and short.

Because immunofluorescent identification of cardiomyocytes on heart sections could be ambiguous [55], we isolated cardiomyocytes from EdU pulse-chased hearts and repeated the analysis. EdU^+^ cardiomyocytes were extremely rare among cardiomyocytes isolated from wildtype hearts, but were easily observed (at a frequency of 7.9–12.6 per 10,000 cardiomyocytes) in the *Asxl2^−/−^* isolates. Microscopic examination and measurement of 70 EdU^+^ cardiomyocytes revealed a distribution of size, morphology, and nucleation status: 38 (54%) of EdU^+^ cardiomyocytes were binuclear, rod-shaped, and with lengths comparable to EdU^−^ cardiomyocytes (Figure 6A). In all the binuclear cells, both nuclei are labeled by EdU. Thirteen (19%) were mononuclear, rod-shaped with lengths comparable to EdU^−^ cardiomyocytes (Figure 6B). The rest, 19 (27%), were mononuclear, spindle-shaped, and shorter than the shortest cardiomyocytes isolated from wildtype hearts (Figure 6C).

### 3.5. The Proliferative Cardiogenic Cells in Asxl2^−/−^ Hearts are Distinct from c-kit^+^ Cardiac Stem Cells and cCFU-Fs

The above data suggest that a fraction of the proliferating interstitial cells detected in 12-week-old *Asx2^−/−^* hearts were cardiogenic, and that the abnormal growth of adult *Asx2^−/−^* hearts is due to production of de novo cardiomyocytes by these cells. The adult heart is known to contain resident cardiogenic cells, such as c-kit^+^ cardiac stem cells (CSCs) [26,29] and cardiac colony forming unit-fibroblasts (cCFU-Fs) [38]. We asked whether the cardiogenic phenomenon in the adult *Asxl2^−/−^* heart is due to activation of either of these cell types. 

**Figure 7:**
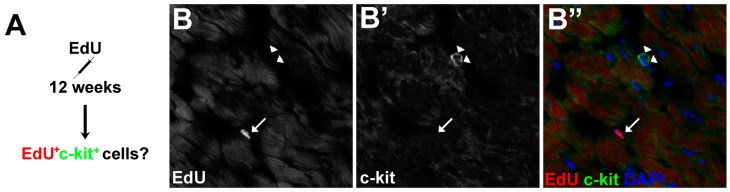
EdU^+^ cells are not c-kit^+^ at 12-weeks of age. (**A**) Wildtype and *Asxl2^−/−^* animals were treated with EdU at 12-weeks of age and frozen sections were assessed for EdU^+^c-kit^+^ cells; (**B**–**B′′**) Representative image of co-labeling for EdU and c-kit. EdU^+^c-kit^+^ cells were not observed. Sample size: *n* = 3 animals per genotype; two non-consecutive sections/heart; ten 20× images/section; 188 wildtype EdU^+^ cells, 265 *Asxl2^−/−^* EdU^+^ cells. Arrows indicate the EdU^+^ nuclei and the arrowheads indicate c-kit^+^ cells.

We pulse labeled proliferative cells in 12-week-old *Asxl2^−/−^* and control hearts with EdU, and co-stained heart sections for EdU and c-kit (Figure 7A). c-kit^+^ cells were readily detected (Figure 7B). However, we did not observe any EdU^+^c-kit^+^ cells, nor was there a higher frequency of c-kit^+^ cells in the *Asxl2^−/−^* heart. Of 188 EdU^+^ cells identified on wildtype heart sections (*n* = 3, two sections/heart) and 265 EdU^+^ cells from *Asxl2^−/−^* sections (*n* = 3, two sections/heart), none was c-kit^+^. cCFU-Fs are marked by a high level of PDGFRα expression and proximity to blood vessels [38]. We examined EdU^+^ cells in pulse-labeled 12-week-old hearts for PDGFRα expression. While many of the EdU^+^ cells express PDGFRα, EdU^+^PDGFRα^high^ cells in *Asxl2^−/−^* hearts are neither more abundant nor more concentrated near blood vessels than those in wildtype hearts (Supplemental Figure S7). These results suggest that the proliferative cardiogenic cells in *Asx2^−/−^* hearts are distinct from c-kit^+^ CSCs and cCFU-Fs.

## 4. Discussion

### 4.1. Adult Asxl2^−/−^ Hearts Exhibit De Novo Cardiomyocyte Production

EdU^+^ cardiomyocytes are readily observed in *Asxl2^−/−^* hearts that are pulse-labeled with EdU at 12 weeks and chased for 4 weeks, but not in similarly pulse-chased wildtype hearts. How did these EdU^+^ cardiomyocytes arise? Proliferation of existing cardiomyocyte is the dominant mechanism for cardiomyocyte production in the adult mammalian heart [5,7]. The majority of studies estimated the rate of cardiomyocyte proliferation to be very low during adulthood, at or below ~1% per year [1,2,4,5,6,7,8,9]. Higher rates of cardiomyocyte proliferation have been reported when the activities of certain genes are manipulated [11,12,13,14,15,16,17,18,19,20,21,22,23,24]. However, this is not the case in *Asxl2^−/−^* mice. While the overall proliferation index is higher in 12-week *Asxl2^−/−^* hearts than in wildtype hearts (Figure 3), the vast majority (~98%) of EdU-labeled cells are non-cardiomyocytes: they are small, express vimentin, and do not express cTnT. Hence, the significantly higher frequency of EdU^+^ cardiomyocytes in *Asxl2^−/−^* hearts after the chase (Figure 5, Supplemental Figure S5) is not due to proliferation, binucleation/polynucleation, or hypertrophic growth of existing cardiomyocytes. Neither did they arise via fusion between an EdU^+^ cell and a pre-existing EdU^−^ cardiomyocytes: EdU^+^ cardiomyocytes isolated from chased *Asxl2^−/−^* hearts are either mononuclear or binuclear with both nuclei labeled by EdU (Figure 6). We did not observe any cardiomyocyte with one EdU^+^ and one EdU^−^ nucleus.

Our evidence strongly suggests that the EdU^+^ cardiomyocytes in pulsed-chased *Asxl2^−/−^* hearts are progenies of non-cardiomyocytes that took up EdU during the time of the pulse label. We hypothesize that *Asxl2^−/−^* hearts harbor a population of proliferative and cardiogenic non-cardiomyocyte (PCN) cells, which can differentiate and produce de novo cardiomyocytes. Consistent with this scenario, EdU^+^ cardiomyocytes isolated from pulse-chased *Asxl2^−/−^* hearts display morphological variations that may be correlated with which stage they are at during the differentiation process. While the largest of them are indistinguishable from mature cardiomyocytes (Figure 6A), the size and morphology of the smaller ones (Figure 6B,C) are suggestive of an intermediate or early stage of differentiation. The definitive testing of our hypothesis awaits the determination of the lineage origin of PCN cells and the genetic labeling and tracing of these cells. 

### 4.2. What Are PCN Cells?

Many questions remain unanswered about PCN cells. First of all, the cellular identity of PCN cells remains unclear. Our evidence suggests that the PCN cells are not c-kit^+^ CSCs, cCFU-Fs, endothelial cells, or ISL1^+^ cardioblasts [26,38,56,57,58]. It has been shown that cardiac fibroblasts can be directly reprogrammed into cardiomyocytes with a trio of transcription factors, GATA4, MEF2C, and TBX5 [39,41]. More recently, increased reprogramming efficiency was reported by using relatively higher levels of MEF2C and lower levels of GATA4 and TBX5 [59]. The majority of freshly labeled EdU^+^ cells are vimentin^+^ and PDGFRα^+^, which are known to be expressed by the fibroblast population. Interestingly, we observed a ~3-fold increase in BrdU^+^MEF2C^+^ cells in un-chased *Asxl2^−/−^* heart. These observations raise the possibility that PCN cells are spontaneously reprogramming fibroblasts. However, we did not detect an increase in EdU^+^GATA4^+^ cells. Moreover, neither vimentin nor PDGFRα exclusively mark fibroblasts [60,61]. Taken together, immunofluorescence-based marker analyses eliminated several scenarios but were not sufficient to pinpoint the identity of PCN cells. A more comprehensive approach is warranted to gain molecular insight into this highly intriguing cell population. While beyond the scope of this paper, a potential starting point could be to determine which transcripts are enriched in EdU^+^ cells isolated from *Asxl2^−/−^* hearts compared to those from wildtype hearts. The correlation between select enriched transcripts and cardiogenic cells in *Asxl2^−/−^* hearts would need to be further examined by genetic lineage tracing and/or by cell isolation followed by in vitro differentiation assays.

Secondly, the exact time frame when PCN cells are active has not been determined. The heart size of *Asxl2^−/−^* mice is proportionally comparable to wildtype up to eight weeks, suggesting that if PCN cells are active in young mice, the activity is too low to have a noticeable effect. Interestingly, ASXL2 is a member of Polycomb Group (PcG) proteins, which are best known for their roles in the longer-term epigenetic maintenance of lineage-specific expression pattern [62]. A number of studies of PcG mutants have reported a deterioration of gene expression pattern over time, after the correct pattern is established [63]. It is conceivable that the loss of ASXL2 resulted in a gradual disruption of gene expression in PCN cells, eventually resulting in their activation. 

Finally, the cardiogenicity of PCN cells remains to be elucidated. In our EdU pulse-chase lineage tracing experiment, ~6% of EdU labeled cells in the *Asxl2^−/−^* left ventricle become cardiomyocytes after a 4-week chase (Figure 5B). In cardiomyocyte isolates from two chased *Asxl2^−/−^* hearts, the frequencies of EdU^+^ cardiomyocytes are 0.079% and 0.126%, respectively. These data, while preliminary, suggest that the cardiogenicity of PCN cells is significantly higher than that reported for c-kit^+^ cells: in a genetic lineage tracing experiment that continuously labels the c-kit lineage from embryonic stage to four weeks after birth, only 0.0027% of cardiomyocytes arise from c-kit^+^ cells [29]. In the future, it will be exciting to fully assess PCN cells’ cardiogenicity by genetic lineage tracing and to determine how PCN cells affect the ability of *Asxl2^−/−^* hearts to regenerate lost muscles in response to myocardial infarction.

### 4.3. Epigenetic Regulation of Cardiogenicity?

Epigenetic factors play crucial roles in the regulation of a cell’s molecular signature and hence its cellular identity [64,65]. The three major epigenetic mechanisms—DNA methylation, histone modification, and chromatin remodeling—center around enzymes that modify DNA, histones, or chromatin organization, respectively. Several drugs that target epigenetic enzymes have been used in cancer treatment with some success [66,67,68]. 

In the heart, multiple epigenetic factors have been shown to regulate transcriptional activities, shape the process of cardiac morphogenesis, and modulate adult cardiac function [69,70,71]. ASXL2 is an essential regulator of histone H2A ubiquitination and H3K27 trimethylation [43,44,72,73]. Our discovery that the *Asxl2^−/−^* heart harbors a population of cardiogenic non-cardiomyocytes suggests that ASXL2 may be part of an epigenetic barrier that prevents a subset of non-cardiomyocytes from adopting a cardiomyocyte fate. Indeed, several recent reports suggest that epigenetic mechanisms are involved in regulating the adult heart’s ability to produce cardiomyocytes both via fibroblast reprogramming and via progenitor differentiation [74,75,76]. These reports, along with our study, raise the exciting possibility of unlocking cardiogenicity in the adult heart by transiently modulating the activities of epigenetic enzymes. For example, if the wildtype heart harbors dormant PCN cells, modulating ASXL2 or its associated epigenetic activities may be an effective way to induce cardiogenic ability in situ, providing an alternative or a complementing approach to existing strategies of heart regeneration. 

## Supplementary Materials

The following are available online at www.mdpi.com/2221-3759/4/4/32/s1.
