# Supplementary material for: Asxl2−/− Mice Exhibit De Novo Cardiomyocyte Production during Adulthood
Source: J Dev Biol. 2016 Nov 3;4(4):32. doi: 10.3390/jdb4040032 (PMC5831801; doi:10.3390/jdb4040032)
Supplement: Supplementary file 1 [file jdb-04-00032-s001.pdf]

Supplementary Materials: *Asxl2*$^{-/-}$ Mice Exhibit De Novo Cardiomyocyte Production during Adulthood

Rachel Brunner, Hsiao-Lei Lai, Zane Deliu, Elan Melman, David L. Geenen and Q. Tian Wang

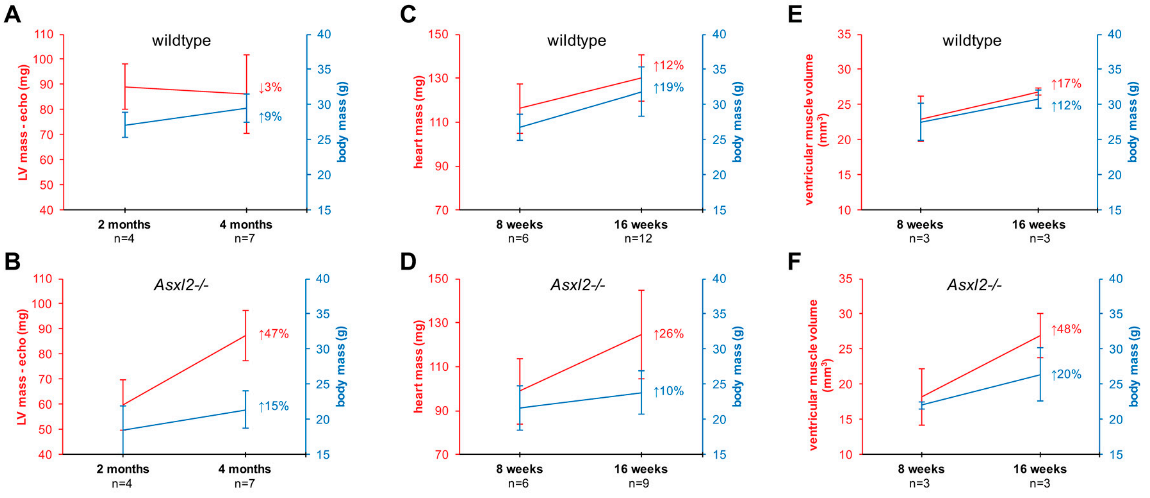

Figure S1. Assessment of cardiac versus body growth in wildtype and *Asxl2*$^{-/-}$ mice. (**A**,**B**) Left ventricular (LV) mass calculated via echocardiography (echo) at two and four months of age in wildtype (**A**) and *Asxl2*$^{-/-}$ (**B**) animals; (**C**,**D**) heart mass of freshly dissected hearts at 8 and 16 weeks of age in wildtype (**C**) and *Asxl2*$^{-/-}$ (**D**) animals; (**E**,**F**) quantitative morphometric analysis of ventricular muscle volume at 8 and 16 weeks of age in wildtype (**E**) and *Asxl2*$^{-/-}$ (**F**). Sample size is shown below the graphs. Bars represent standard deviation.

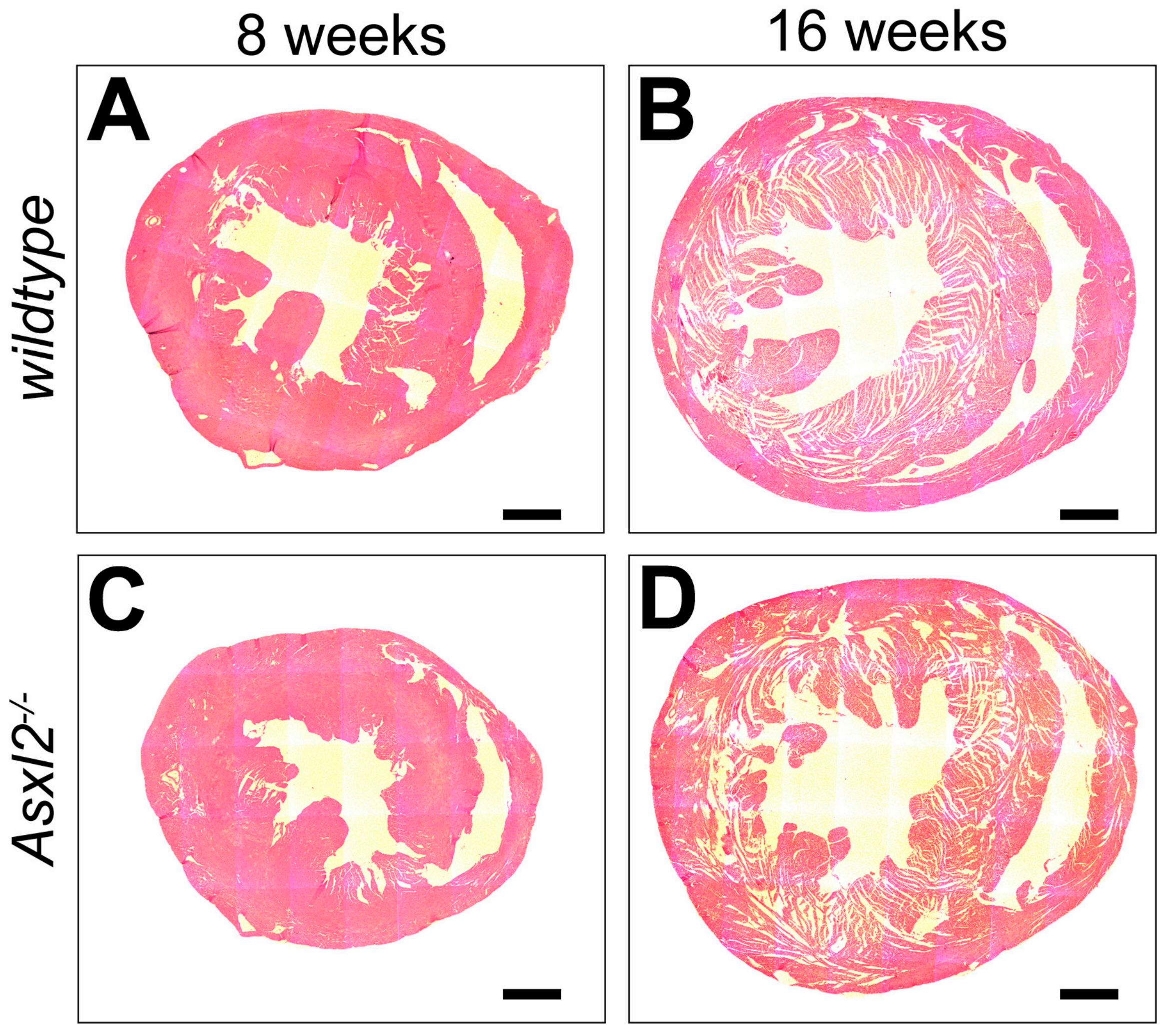

Figure S2. Histological examination of *Asxl2*$^{-/-}$ hearts. Shown are representative hematoxylin and eosin stained cross-sections from (**A**) wildtype heart at 8 weeks of age; (**B**) wildtype heart at 16 weeks of age; (**C**) *Asxl2*$^{-/-}$ heart at 8 weeks of age; and (**D**) *Asxl2*$^{-/-}$ heart at 16 weeks of age. Body masses of mice with heart cross-sections represented here are (**A**) 24.5 grams; (**B**) 30.0 grams; (**C**) 22.5 grams; (**D**) 28.6 grams. Scale bar = 500 µm.

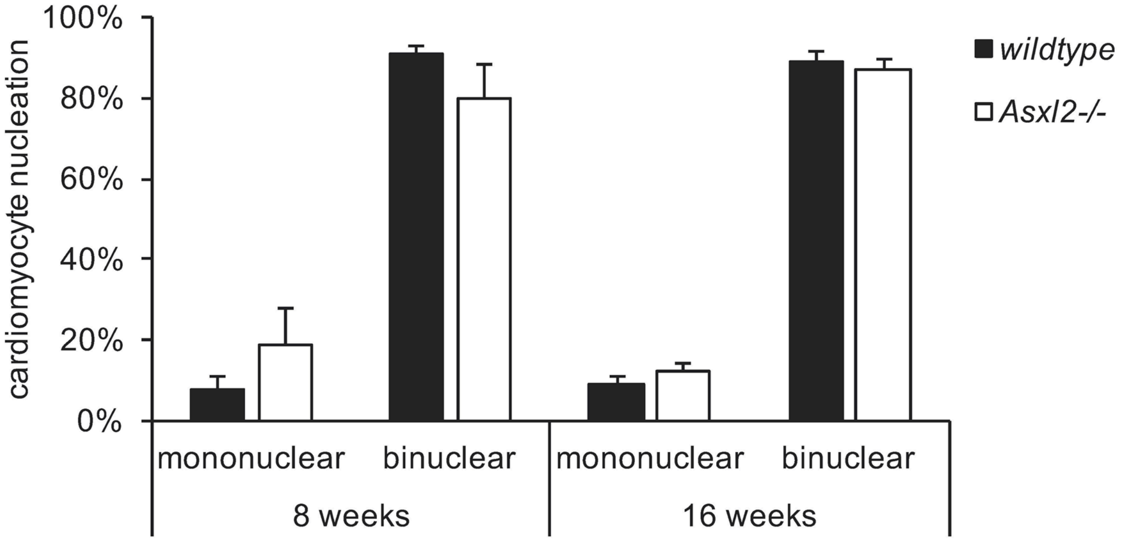

Figure S3. Analysis of cardiomyocyte nucleation status in *Asxl2*$^{-/-}$ and wildtype hearts. Cardiomyocytes were isolated from 8- and 16-week wildtype (8-week time-point: n = 4; 16-week time-point: n = 5) and *Asxl2*$^{-/-}$ (n = 3 per time-point) hearts and assessed for number of nuclei per cardiomyocyte. Bars represent standard deviation. No significant differences were observed (Student's t-test).

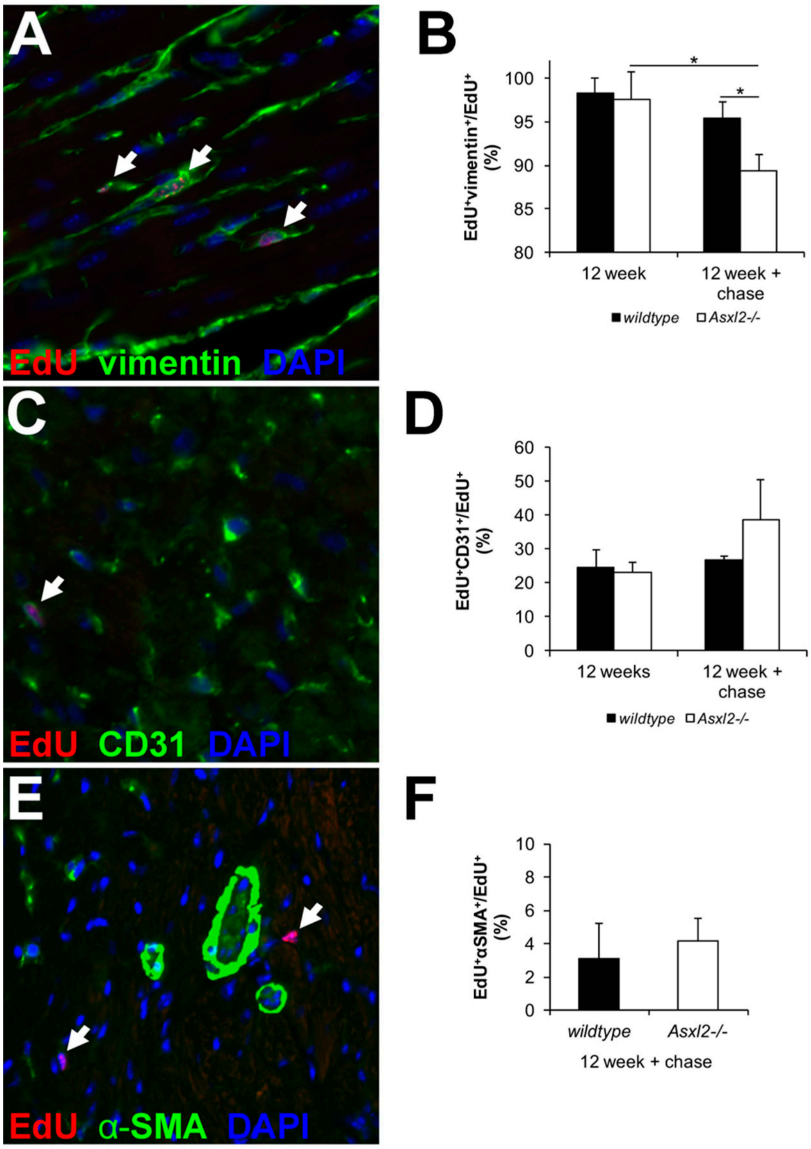

Figure S4. Analysis of vimentin, CD31, and α-SMA expression among EdU⁺ cells in chased *Asxl2*$^{-/-}$ hearts. (**A**) Representative image of EdU, DAPI, and vimentin labeling; (**B**) quantification of percentage of EdU⁺ cells positive for vimentin; (**C**) Representative image of EdU, DAPI, and CD31 labeling; (**D**) quantification of percentage of EdU⁺ cells positive for CD31; (**E**) Representative image of EdU, DAPI, and α-SMA; (**F**) quantification of percentage of EdU⁺ cells positive for α-SMA. At least three non-consecutive sections, 25 images/section, from three animals per genotype/timepoint were assessed. Arrows indicate EdU⁺ nuclei. Bars represent standard deviation. * *p*-Value (Student's *t*-test) < 0.05.

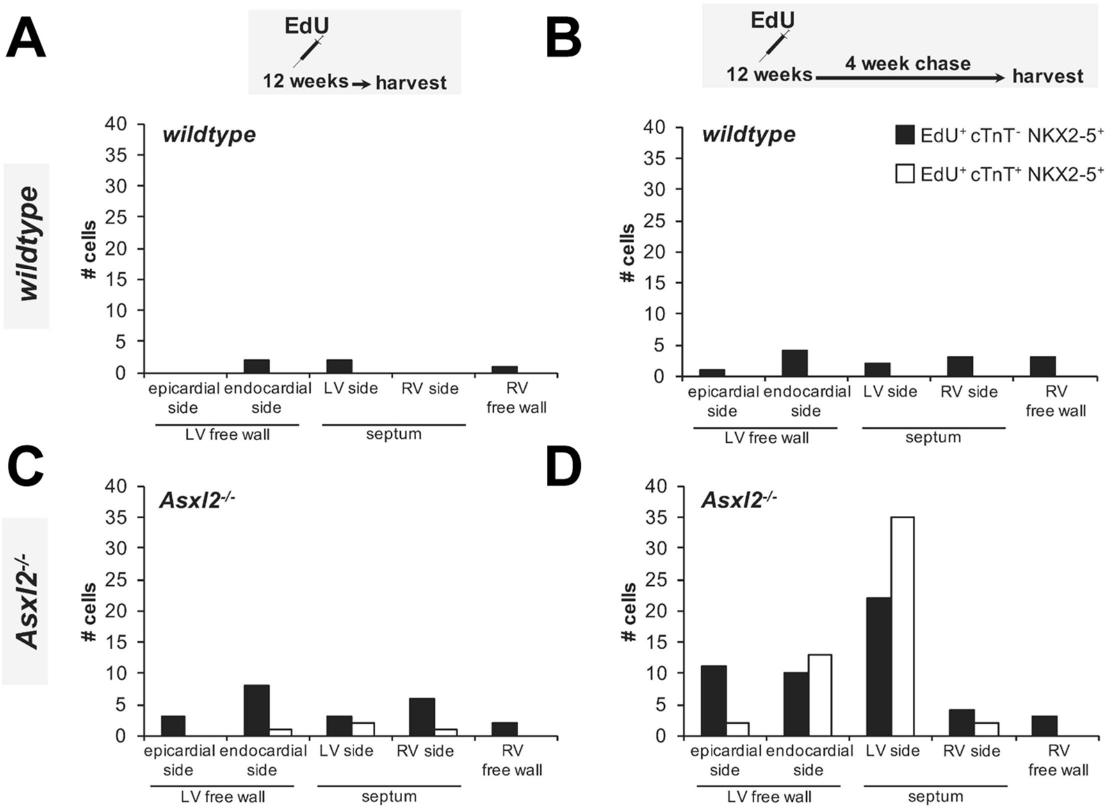

Figure S5. Number and distribution of EdU⁺cTnT⁻NKX2-5⁺ and EdU⁺cTnT⁺NKX2-5⁺ cells in unchased (**A**) and chased (**B**) wildtype, and unchased (**C**) and chased (**D**) *Asxl2*$^{-/-}$ hearts. EdU⁺ cells were classified according to whether they were cTnT⁻NKX2-5⁺ (black bars), cTnT⁺NKX2-5⁺ (white bars), or cTnT⁻ NKX2-5⁻ (not shown). Sample size: n = 3 animals per genotype per scheme (unchased vs. chased); three non-consecutive sections/heart. Five 20× images/specific location per section were analyzed in unchased hearts. Whole sections (stitched from 20× images in ZenPro software) were analyzed in chased hearts.

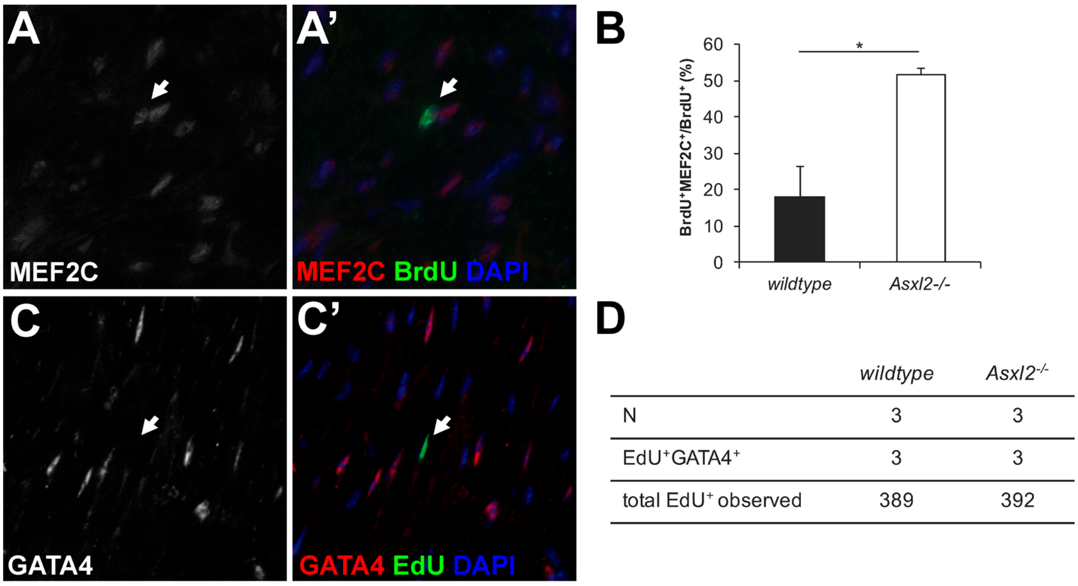

	wildtype	Asxl2-/-
N	3	3
EdU+GATA4+	3	3
total EdU+ observed	389	392

Figure S6. Expression of the cardiogenic markers MEF2C and GATA4 among BrdU+ or EdU+ cells in 12-week hearts. (**A**,**A'**) Representative image of BrdU and Mef2C labeling; (**B**) Quantification of the percentage of BrdU+Mef2C+ cells in the left ventricle (n = 3 per genotype, three non-consecutive sections/heart, twenty-five 20× images/section); (**C**,**C'**) Representative image of EdU and GATA-4 labeling; (**D**) EdU+GATA4+ cells in the left ventricles are rare in both the wildtype and *Asxl2*–/–. Bars represent standard deviation. * p-Value (Student's t-test) < 0.05.

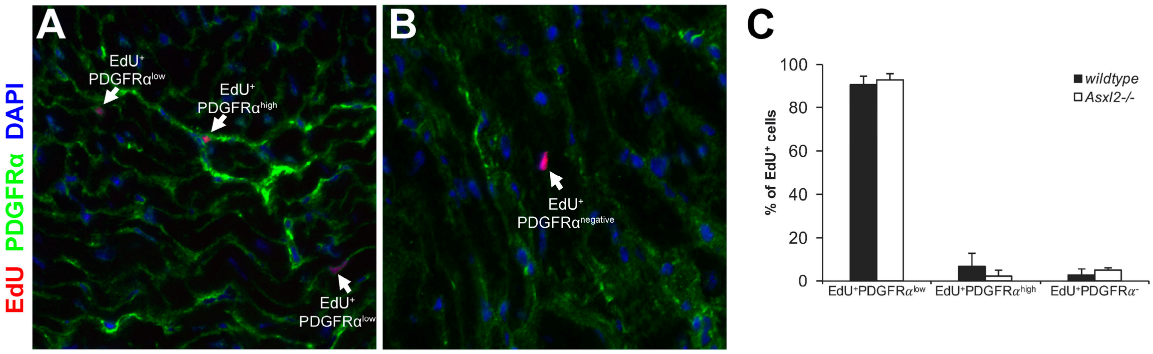

Figure S7. Examination of PDGFRα expression among EdU+ cells in 12-week hearts. (**A**,**B**) Representative images from frozen sections of EdU, anti-PDGFRα, and DAPI labeling; Overall, most small cells were positive for low levels of PDGFRα and many cells near blood vessels had high levels of PDGFRα, consistent with a previous report (Chong et al. 2011); (**C**) Quantification of the percentage of EdU+ cells that had low or high levels, as well as those that were negative for, PDGFRα. At least three non-consecutive sections from three animals per genotype were assessed. Bars represent standard deviation.
